# *BAG2*, *MAD2L1*, and *MDK* are cancer-driver genes and candidate targets for novel therapies in malignant pleural mesothelioma

**DOI:** 10.1038/s41417-024-00805-4

**Published:** 2024-09-12

**Authors:** Luisa Bisceglia, Federica Morani, Lara Guerrieri, Eric Santoni-Rugiu, Pınar Çakılkaya, Cristian Scatena, Rosa Scarpitta, Lars H. Engelholm, Niels Behrendt, Federica Gemignani, Stefano Landi

**Affiliations:** 1 Department of Biology, University of Pisa, Pisa, Italy; 2 Department of Pathology, Rigshospitalet, University of Copenhagen, 2100 Copenhagen, Denmark; 3 Department of Clinical Medicine, University of Copenhagen, 2200 Copenhagen N, Denmark; 4 Finsen Laboratory, Rigshospitalet/Biotech Research & Innovation Centre (BRIC), University of Copenhagen, 2200 Copenhagen, Denmark; 5 Dipartimento di Ricerca Traslazionale e delle Nuove Tecnologie in Medicina e Chirurgia, Università di Pisa, Pisa, Italy; 6 UO Anatomia Patologica 1 Universitaria, DAI – Medicina di Laboratorio, Azienda Ospedaliero Universitaria Pisana, Pisa, Italy

**Keywords:** Mesothelioma, Cancer genetics

## Abstract

Malignant pleural mesothelioma (MPM) is an aggressive cancer with a poor prognosis and the identification of novel druggable targets is urgently needed. In previous work, we identified 15 deregulated genes highly expressed in MPM tissues and correlated with a poor prognosis. Here, we validated these findings on an independent dataset of 211 MPM patients (EGA, EGAD00001001915) and on a panel of MPM cell lines. Furthermore, we carried out in vitro gene silencing followed by proliferation, cytotoxicity, caspase, and migration assays to define whether these targets could be cancer-driver genes. We ended up with three novel candidates (i.e., *BAG2*, *MAD2L1*, and *MDK*), whose encoded proteins could be exploited as druggable targets. Moreover, of novelty, immunohistochemistry analysis on tissues revealed that the overexpression of BAG2 and MAD2L1 could differentiate MPM from RMP patients. Furthermore, when we tested Neratinib (an inhibitor of MAD2L1) and iMDK (an inhibitor of MDK) we found that they are effective on MPM cells, in part phenocopying the effects of *MAD2L1* and *MDK* gene silencing. In summary, in the present work, we report that *BAG2*, *MAD2L1*, and *MDK* are bona fide cancer-driver genes for MPM worth of further studies.

## Introduction

Malignant pleural mesothelioma (MPM) is a rare incurable cancer triggered by asbestos exposure [1]. Because of its unspecific symptoms, patients are not promptly diagnosed delaying the only available chemotherapies consisting of platinum-based compounds plus pemetrexed [2–5]. The recent introduction of immune checkpoint inhibitors has shown some success in a fraction of patients [6, 7], therefore the identification of novel druggable targets in MPM remains an urgent need.

Among various mechanisms, cancer progression can be driven by the aberrant overexpression of proteins encoded by cancer-driver genes (CDGs). Following this idea, the inhibition of such abundantly expressed proteins could constitute a valid therapeutic approach. To reach this goal, one of the first steps is the identification of genes expressed at high levels in MPM. However, differentially expressed genes (DEGs) could be observed also as an epiphenomenon, i.e., as a consequence of the phenotypic alterations occurring in malignant cells. These DEGs are not truly CDGs, they should be more correctly defined as “response genes” (RGs). Analysis of the correlation between increased DEGs expression and reduced OS in case-control studies could be another criterion for refining candidate CDGs list. Based on these concepts, in a previous study, we carried out bioinformatics analyses on public databases detecting 15 DEGs overexpressed in MPM and whose extent of overexpression was also inversely correlated with patients’ OS [8].

The proteins encoded by these genes, namely, BAG2, CIT, CTHRC1, E-selectin, MAD2L1, Midkine (MDK), PODXL, SPARC, TNNT1, TRAF2, UHRF1, ADAMTS1, DSC3, KIF23, and PRSS23 were confirmed in MPM cell lines [8]. The fact that a gene shows a high expression in primary tumors as well as in patient-derived permanent cell lines could be due to its relevance in sustaining the growth of these malignant cells. Thus, the results of this screening further corroborated the idea that these DEGs could be bona fide CDGs.

However, a further criterion for sorting CDGs from RGs is to evaluate in malignant cell lines the phenotypic effects when the abundantly expressed protein undergoes to gene silencing. It is conceivable that a reduction of the levels of these proteins could modulate some of the malignant hallmarks eliciting, for example, reduced proliferation or increased apoptosis.

The goal of the present study was to verify, both in silico and with functional assays, whether the previously identified DEGs could be bona fide CDGs to be exploited as therapeutic targets. First, DEGs were validated on an independent transcriptomic dataset. Then, we performed in vitro assays ending with three highly likely CDGs: *BAG2*, *MAD2L1*, and *MDK*. The expression of these proteins was further verified and confirmed by immunohistochemistry (IHC) methods on a series of primary MPM tissues.

## Materials and methods

### Databases, data processing, and expression analysis

The RNA-seq dataset from the European Genome-phenome Archive EGAD00001001915 (https://ega-archive.org/datasets/EGAD00001001915; 17/12/2021), including 141 epithelioid (EMM), 62 biphasic (BMM), 7 sarcomatoid (SMM), and 1 desmoplastic (DMM) mesotheliomas, was used for validation. The DepMap database was accessed via https://depmap.org/portal/ in 09/2022. RNA-seq samples of EGA and Gene Expression Omnibus (GEO) were processed/reprocessed using the same pipeline described in the Genomic Data Commons Data Portal–The Cancer Genome Atlas (GDC–TCGA) (https://portal.gdc.cancer.gov/; 25/03/2020). In brief, the downloaded data were analyzed with FASTQC [9], then the processed reads were mapped to the human genome (GRCh38.d1.vd1) using STAR [10]. GEO raw counts were calculated using HTSeq [11], while EGA raw counts were obtained using STAR. The gene IDs were annotated using the biomaRt package [12]. The raw counts for the 211 MPM and 3 normal lung specimens were used as inputs for DESeq2 [13]. Within the outputs, we verified the 15 DEGs previously identified [8]. All data were handled using R (https://www.R-project.org ; 15/03/2020).

### Survival analysis

Patients were stratified as high/low groups based on the median of each gene expression. Kaplan–Meier survival estimators were used to analyze each patient group’s prognosis, and log-rank tests were used to compare the two groups’ survival results. The R packages survival and ggplot2 [14] were used to calculate the log-rank and plot the curves. Average and SD are displayed in the box plots. After confirming that the assumptions were met (*p* > 0.05), two-way analysis of variance (ANOVA) and the Tukey multiple comparison test were employed to compare means across the groups (***p value < 0.001; **p value < 0.01; *p value < 0.05, ns *p* value > 0.05).

### Cell culture

MeT-5A (RRID:CVCL_3749) and MSTO-211H (RRID:CVCL_1430) cells were purchased from American Type Culture Collection (ATCC, Manassas, VA, USA), authenticated within the last 3 years with short tandem repeat profiling, and cultured in mycoplasma-free conditions with 100 U/mL penicillin and 100 U/mL streptomycin (Euroclone S.p.A., Milan, Italy) at 37 °C, 5% CO_2_ atmosphere. For Met-5A the Medium 199 was supplemented with 10% FBS, 3.3 nM EGF, 400 nM hydrocortisone, and 870 nM zinc-free bovine insulin (Gibco, Carlsbad, CA, USA). For MSTO the 1:1 mixture DMEM/Ham’s F-12 was supplemented with 15% FBS and 2 mM l-glutamine (Euroclone S.p.A., Milan, Italy).

### Western blot (WB) analysis

Cells were collected at confluence, washed twice in PBS, and homogenized in Mammalian Protein Extraction Reagent (M-PER; ThermoFisher Scientific, Waltham, MA, USA) containing inhibitors of proteases and phosphatases (Roche Diagnostics GmbH, Rotkreuz, Switzerland). The extracted proteins were quantified by BCA test (Carlsbad, CA, USA) and 7 μg were denatured and separated by electrophoresis using precast Novex 8–16% or 4–12% Wedge Wells Tris-Glycine Gels (Invitrogen-Life Technologies, Carlsbad, CA, USA) and electroblotted onto PVDF membranes (Bio-Rad Laboratories Inc., Hercules, CA, USA). The membranes were blocked with 5% milk in TBST and probed overnight at 4 °C with the specific rabbit polyclonal antibodies (Abs) against BAG2 (cod A304-751A-T, 1:1000; Bethyl Laboratories Inc., Montgomery, TX, USA), MAD2L1 (cod 10337-1-AP, 1:800), Midkine (cod 28546-1-AP, 1:500), β-tubulin (cod 10068-1-AP, 1:8000), as well as with the mouse monoclonal Abs against epidermal growth factor receptor (EGFR) (cod 18986-1-A, P, 1:7000) and GAPDH (cod 60004-1-Ig, 1:10,000) (all from Proteintech, Rosemont, IL, USA). β-tubulin was used as the index of protein loading in the lanes. The secondary Abs, anti-rabbit IgG-HRP (cod 111-035-144, 1:10,000; Jackson ImmunoResearch Laboratories, West Grove, PA, USA) and anti-mouse IgG-HRP (cod SA00001-1, 1:20,000; Proteintech, Rosemont, IL, USA) were added for 1 h at room temperature and used for signal detection. Reactive bands were detected using Clarity MaxTM Western ECL Substrate (Bio-Rad Laboratories Inc., Hercules, CA, USA). Visualization was performed using a ChemiDoc Imaging System (Bio-Rad Laboratories Inc., Hercules, CA, USA). Densitometry of bands was carried out with ImageLab 6.0 software (Bio-Rad Laboratories Inc., Hercules, CA, USA).

### Gene silencing or drug treatments

MSTO and Met-5A cells were seeded at optimized densities in 6-well or 96-well plates. At 24 h cells reached 60–80% confluence, the medium was substituted and enriched with the transfection mix (Lipofectamine RNAiMax, Invitrogen-Life Technologies, Carlsbad, CA, USA; reduced serum medium Opti-MEM, Gibco, Carlsbad, CA, USA; the chosen siRNA, 10 µM). All siRNAs were SMARTpool ON-TARGETplus siRNA (Dharmacon Inc., Lafayette, CO, USA). A non-targeting siRNA-negative-control for non-specific siRNA delivery effects (a baseline for target gene silencing), and the positive-control-siRNA targeting GAPDH were employed. Cells in 96-well plates were placed in IncuCyte system (Sartorius AG, Goettingen, Germany). This is the *t*_0_ time for the live imaging analysis, which will last for a further 72 h. Cells in 6-well plates were placed in the incubator and collected after 72 h for protein extraction, WB analysis, and verification of gene silencing that was considered successful when the target protein showed >60% of reduced expression. For drug treatments, MSTO and MeT-5A cells were seeded in 96-well plates at appropriate densities and after 24 h (30–50% confluence) treated with iMDK (7.5, 15, and 50 µM) or Neratinib (10, 20, and 40 µM) (Tocris Bioscience, Bristol, UK), dissolved in DMSO, or with the vehicle alone. The treatments lasted until the end of the assays.

### Cell proliferation assay and cell viability

Silenced/treated cells were monitored with IncuCyte® live-cell imaging for 72 h. Cell growth was measured by analyzing the area of confluence, normalized to *t*_0_. At 72 h after transfection cells were rinsed twice with serum-free media HBSS (Gibco, Carlsbad, CA, USA). Calcein AM (Invitrogen-Life Technologies, Carlsbad, CA, USA), 2 µM in HBSS, was added to the cells and incubated for 2 h. Plates were placed in the IncuCyte for 2 h and the images were analyzed using IncuCyte Base Analysis Software (Sartorius AG, Goettingen, Germany) where fluorescence intensity (FI) was proportional to the number of living cells. At the end, the overall FI was also read with FLUOstar Optima (BMG Labtech, Ortenberg, Germany) plate reader (at 494/517 nm).

The Shapiro–Wilk test was used to verify the normal distribution of the data, from at least three repeats. If there was a statistically significant difference between groups, contrasts were evaluated with ANOVA or multiple Mann–Whitney tests. The statistical analysis was carried out by using GraphPad Prism 9 software (GraphPad Software, Inc., San Diego, CA, USA).

### Caspase 3/7 and cytotoxicity assay

Cultures were enriched with IncuCyte® Caspase 3/7 Green Dye at 5 µM or the IncuCyte® Cytotox Red Dye at 25 nM (all from Sartorius AG, Goettingen, Germany). Then, 96-well plates were placed in IncuCyte instrument for 72 h and the collected images were analyzed using IncuCyte Base Analysis Software (Sartorius AG, Goettingen, Germany). To compare the median values of the different treatment groups, we performed multiple Mann–Whitney tests.

### Wound healing assay

MSTO and MeT-5A cells were seeded in 96-well IncuCyte ImageLock Plates and grown to confluence. After 24 h a cell-free gap (scratch) in each well was made with IncuCyte Wound Maker 96-Tool (Sartorius AG, Goettingen, Germany). Then, cells were washed with PBS and treated with different combinations of siRNAs or drugs, using low serum concentrations in cell medium (2% FBS) to suppress cell proliferation. The scratch areas were monitored for 24 h, using a phase-contrast setting in the IncuCyte instrument. Images were captured every 3 h. The percentage of wound closure (WC) was calculated using the following formula:

$${{WC}}=\frac{{{area}}_{t0}-{{area}}_{{ti}}}{{{area}}_{t0}}\times 100$$

We employed a simple linear regression to examine the relationship between WC over time. Subsequently, statistical analysis, including ANOVA followed by Sidak’s multiple comparison tests, was conducted. Prior to analysis, we ensured the fulfillment of assumptions.

### Formalin-fixed paraffin-embedded (FFPE) tissue specimens

Forty FFPE archival MPM tissue specimens were collected (in 2017–2020) from 28 patients at the Department of Thoracic Surgery and analyzed at the Department of Pathology of Rigshospitalet, Copenhagen University Hospital, Denmark [15]. Signed informed consent was obtained from all patients. Twelve patients presented matched biopsies + resections, eight (inoperable) had only biopsies, and eight were only resections. The histologic subtypes were 12 EMM, 17 BMM, 6 SMM, and 5 DMM. All patients who underwent pleurectomy/decortication had EMM or BMM and had received three cycles of standard neoadjuvant chemotherapy with cisplatin-pemetrexed doublet. Five patients diagnosed with BMM showed a transformation to DMM in the corresponding P/D. For each specimen, only tissue sections with a tumor cell content >50% were used. As non-neoplastic controls, we used 13 FFPE tissue samples from 12 patients with reactive mesothelial proliferation (RMP), who had been operated for benign intrathoracic pathologies not related to MPM (6 spontaneous pneumothorax, 4 lung fibrosis, 1 post-empyema pleural fibrosis, and 1 aorta aneurism).

### Immunohistochemistry (IHC)

IHC was carried out on 3.5 μm paraffin sections by using recombinant Anti-BAG2 Rabbit mAb [EPR3567] (1:400), Anti-Midkine Rabbit mAb [EP1143Y] (1:100; cod ab79406, ab52637; Abcam, Cambridge, MA, USA), and Anti-MAD2 (MAD2L1) Rabbit pAb (1:250; cod TA308923; OriGene, Rockville, MD, USA). The sections were heated for 60 min and deparaffinized using Tissue Clear (Sakura Finetek, Alphen aan den Rijn, Netherlands) and an ethanol gradient. Antigen retrieval treatment was carried out for 15 min at 98 °C with citrate buffer (10 mM Tris, 0.5 mM EGTA, pH 6.0) for the anti-MDK Ab, and TEG buffer (10 mM Tris, 0.5 mM EGTA, pH 9.0) for the anti-MAD2L1 and anti-BAG2 Abs. Then, sections were washed and incubated for 15 min in a solution of Milli-Q water and H_2_O_2_ at 1% (Merck KGaA, Darmstadt, Hesse, Germany) followed by incubation with the specific primary Ab diluted in Antibody Diluent, Background Reducing (Dako, Agilent Technologies, Inc, Santa Clara, California, USA) overnight at 4 °C. After 24 h, sections were washed in TBST (50 mM Tris, 150 mM NaCl, 0.5% Triton X-100, pH 7.6), incubated for 45 min with the secondary Ab EnVision Rabbit, (Dako, Agilent Technologies, Inc, Santa Clara, CA, USA), washed with TBST, and incubated for 5 min with Liquid DAB+ Substrate Kit (2 components for IHC; OriGene, Rockville, MD, USA). Sections were then counterstained for 30 s using Mayer’s hematoxylin (Sakura Finetek, Alphen aan den Rijn, Netherlands). Sections not incubated with primary Abs served as negative controls. Sections were scanned using NDP.view2 Plus software and a NanoZoomer-XR Digital slide scanner C12000-01 (Hamamatsu Photonics, Hamamatsu, Japan). A blinded evaluation of the scans was made by an experienced pathologist (ESR). For each section, H-score was calculated, measuring the percentage of the area exhibiting the different intensities (weak = A; moderate = B; strong = C) at 200× magnification (final score from 0 to 300), as follows [15–17]:

$${{Hscore}}=1{\rm{x}}\left[ \%\, {{cells}}\,{{A}}\right]+2{\rm{x}}\left[ \%\, {{cells}\; B}\right]+3{\rm{x}}\left[ \% \,{{cells}\; C}\right]$$

For statistical purposes, DMMs were merged with SMM patients and Welch’s ANOVA tests corrected for Dunnett T3 were performed. For the evaluation of the IHC scores as biomarkers, the sensitivity, specificity, Yuden index, and the area under the curve (AUC) were calculated with the online tool of the Johns Hopkins University (http://www.rad.jhmi.edu/jeng/javarad/roc/JROCFITi.html).

For IHC of HER2, MSTO cells were FFPE. IHC from breast ductal carcinoma in situ tissue was used as a positive control. For antigen retrieval, heat induction with EDTA buffer (pH 7.4) was used. An automated system (Ventana Benchmark Ultra, Roche Diagnostics, Basel, Switzerland) was used to apply Ventana anti-HER2/neu antibody (clone 4B5, rabbit monoclonal) in accordance with the manufacturer’s instructions.

## Results

### Validation of previous candidate targets using an independent dataset

First, we found that (except for *TRAF2*) all the considered DEGs [8] were upregulated (*p* adjusted < 0.05 and |log2fc| > 0.38) also in EGAD00001001915 [18], and the intensity of their expression was also inversely correlated with the prognosis (especially for *BAG2*, *CIT*, *KIF23*, *MAD2L1*, *MDK*, *PODXL*, *PRSS23*, *SPARC*, *TNNT1*, and *UHRF1*; log-rank test *p* value < 0.05), strikingly confirming our previous work [8]. These analyses are reported in Supplementary Figs. S1 and S2 and in Table 1.

**Table 1:** Scores of the signature genes resulted from the differential expression analysis using as input the EGA RNA-seq data compared to the three lung samples.

Symbol	ID	baseMean	log2FoldChange	lfcSE	stat	padj
*ADAMTS1*	ENSG00000154734	5512.78	2.2	0.74	2.98	0.0087
*BAG2*	ENSG00000112208	1405.02	1.55	0.45	3.47	0.0019
*CIT*	ENSG00000122966	842.21	2.45	0.52	4.69	<0.0001
*CTHRC1*	ENSG00000164932	5541.38	6.3	0.8	7.9	<0.0001
*DSC3*	ENSG00000134762	2331.75	6.9	1.29	5.34	<0.0001
*KIF23*	ENSG00000137807	1608.23	2.38	0.61	3.89	0.0004
*MAD2L1*	ENSG00000164109	756.47	4.65	0.58	8.05	<0.0001
*MDK*	ENSG00000110492	7956.64	6.41	0.74	8.68	<0.0001
*PODXL*	ENSG00000128567	15153.48	3.84	0.86	4.45	<0.0001
*PRSS23*	ENSG00000150687	13813.5	5.08	0.73	6.92	<0.0001
*SELE*	ENSG00000007908	392.77	6.33	1.4	4.51	<0.0001
*SPARC*	ENSG00000113140	170295.4	17.55	1.31	13.35	<0.0001
*TNNT1*	ENSG00000105048	1284.58	9.53	1.63	5.84	<0.0001
*TRAF2*	ENSG00000127191	994.46	0.56	0.37	1.5	0.2501
*UHRF1*	ENSG00000276043	767.47	5.1	0.71	7.22	<0.0001

*Symbol* symbol of the genes, *ID* ensemble ID, *baseMean* average of the normalized count values, dividing by size factors, taken over all samples, *log2FoldChange* effect size estimate, *lfcSE* standard error estimate for the log2 fold change estimate, *stat* Wald statistics, *padj* BH-adjusted *p* values.

**Fig. 1:**
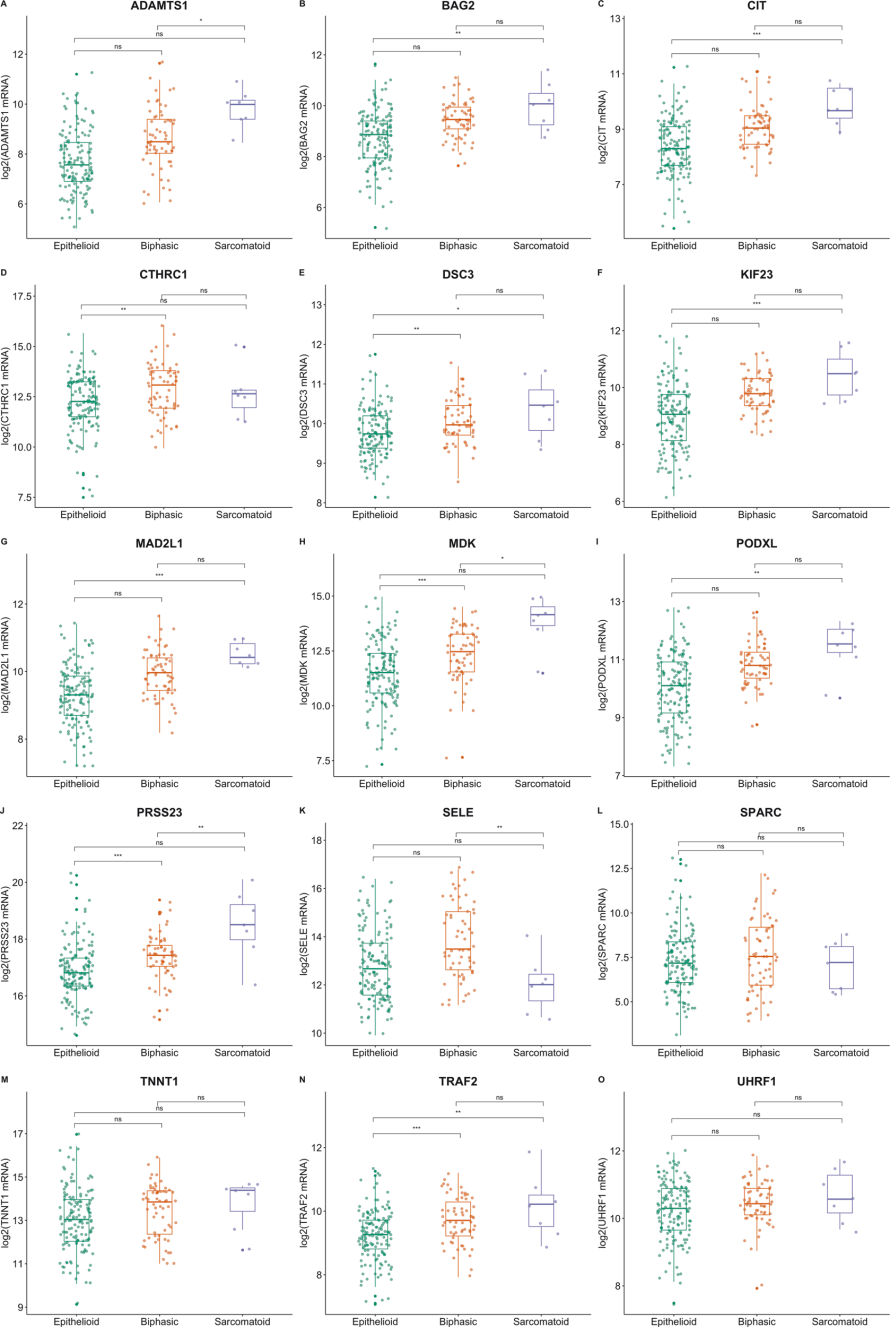
Box plots of the patients stratified by histology. **A**–**O** In green the epithelioid, in orange the biphasic, and in purple the sarcomatoid MPM cases. *p* value = 0.0332(*), 0.0021(**), 0.0002(***).

**Fig. 2:**
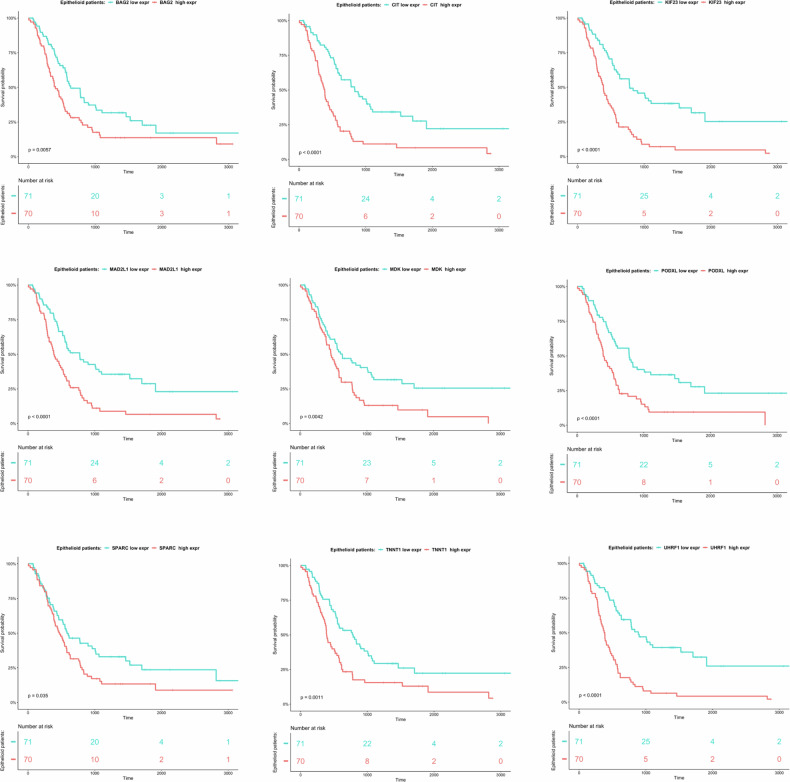
KM only on the epithelioid MPM cases. In red the high-expression group, in blue the low-expression group. The time is expressed in days. Log-rank test was used to calculate the significant difference between the two curves.

SMM and BMM subtypes expressed a higher level of *BAG2, CIT, CTHRC1, DSC3, KIF23, MAD2L1, MDK, PODXL*, *PRSS23*, and *TRAF2* compared to those with the epithelioid (Fig. 1). However, when KM plots analysis was performed stratified for EMM (the largest group with a better prognosis [19]) a statistically significant inverse correlation of the expression of *BAG2, CIT, KIF23, MAD2L1, MDK, PODXL, SPARC, TNNT1*, and *UHRF1* (but not *PRSS23*) with the OS (Fig. 2) was found. Therefore, we considered these nine DEGs as the strongest candidate CDGs.

### Preliminary target selection and refinement

**Fig. 3:**
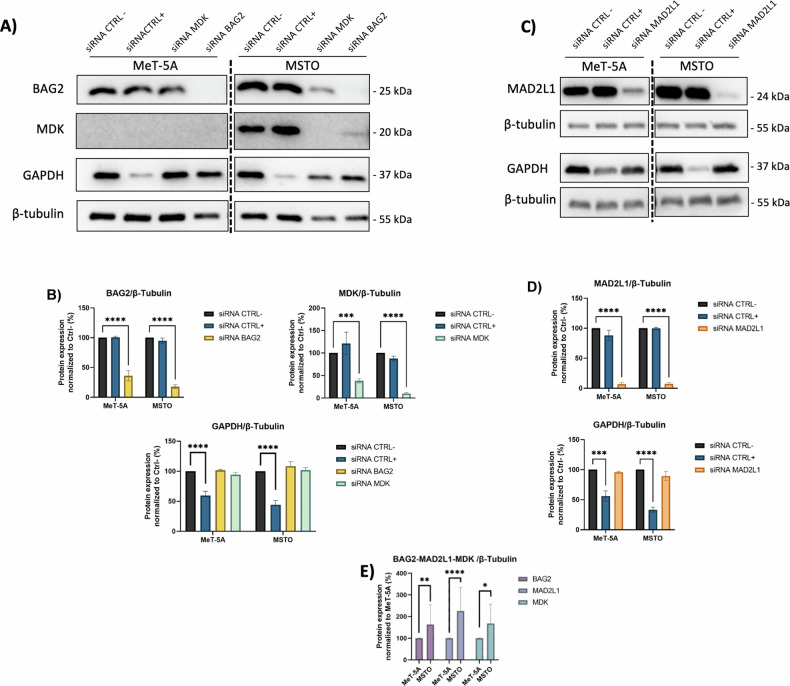
A representative western blot of the siRNA transfection experiment. **A** The blot of siRNA BAG2 and MDK, **C** the blot of siRNA MAD2L1. β-Tubulin was used as a loading control and GAPDH was the positive control of the siRNA transfection. As shown in the blots and also in graphs (**B**) and (**D**) there was a significant reduction of the protein level in the silenced cells, as compared to the negative CTRL. **E** Graph showing the comparison of protein expression in MeT-5A and MSTO cell lines of BAG2, MAD2L1, and MDK (Mann–Whitney test; *p* value < 0.05 were considered significant, *p* value = 0.0332(*), 0.0021(**), 0.0002(***), <0.0001(****)).

Among these candidates, we did not proceed further with *UHRF1* and *SPARC* as these genes have been thoroughly studied and their role as CDGs in MPM has been clearly proved [20, 21]. Moreover, the cell models employed in this work (MeT-5A and MSTO) did not allow to study KIF23 [8], thus, we evaluated BAG2, CIT, MAD2L1, MDK, PODXL, and TNNT1 proteins. We used specific siRNA pools against these DEGs and WB analysis confirmed a strongly reduced expression of BAG2, MAD2L1, and MDK after 72 h of siRNA transfection (Fig. 3). Thus, these proteins were considered lead targets for additional in vitro studies. The remaining genes, despite several attempts of optimization, were not successfully silenced (the residual expression was >60%), therefore, in the present work, they were not considered further, postponing them to future studies when a more efficient way of knocking them down will be approached.

### In vitro functional assays after gene silencing

**Fig. 4:**
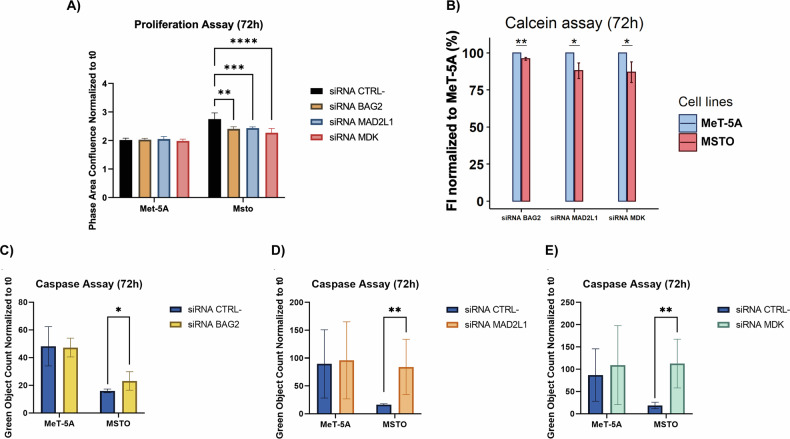
Viability, proliferation and caspase assays after gene silencing. **A** AOC of MeT-5A and MSTO at 72 h after treatment with siRNA-negative-control (siRNA CTRL−), siRNA BAG2, siRNA MAD2L1, siRNA MDK. The AOC is normalized to the time of transfection (*t*_0_). **B** Overall, FI of MeT-5A and MSTO was measured at 72 h after silencing; the graphs show the comparison between MeT-5A and MSTO for the three-siRNA transfection. ANOVA test; *p* value = 0.0332(*), 0.0021(**), 0.0002(***), <0.0001(****). **C**–**E** End point of apoptosis analysis in MSTO and MeT-5A cells in the presence of 5 µM caspase 3/7 Green Dye and siRNA BAG2, siRNA MAD2L1, and siRNA MDK, respectively. Mann–Whitney test; *p* value 0.0332(*), 0.0021(**), 0.0002(***), <0.0001(****).

We tested whether BAG2, MAD2L1, and MDK could play a role in sustaining the viability of MSTO cells. Therefore, we carried out a proliferation assay, measuring the AOC (Fig. 4A) and the FI (Fig. 4B), at 72 h after siRNA transfection. We observed a statistically significant reduction of the viability in the transfected cells compared to the cells treated with the negative-control-siRNA. Interestingly, the reduction was restricted only to MSTO and not to MeT-5A cells. Moreover, the activation of caspases 3/7 was assayed as a proxy for the apoptotic response. We cultured MeT-5A and MSTO cells in the presence of IncuCyte® caspase 3/7 reagent in combination with the siRNA pools targeting either *MDK*, *BAG2*, *MAD2L1*, or the negative control. Caspase 3/7 activation was monitored for 72 h after siRNA transfection, then measured with the IncuCyte software by counting green objects per well, and a statistically significant increase was observed for all the genes (Fig. 4C–E) in MSTO cells. Finally, we measured the ability of cells to migrate, as this behavior is an important hallmark of cancer cells. The wound healing assay was performed in cells transfected with siRNA pools or negative-control-siRNA but we did not observe any statistically significant difference, as reported in Supplementary Fig. S3.

### In vitro functional assays after drug treatment

**Fig. 5:**
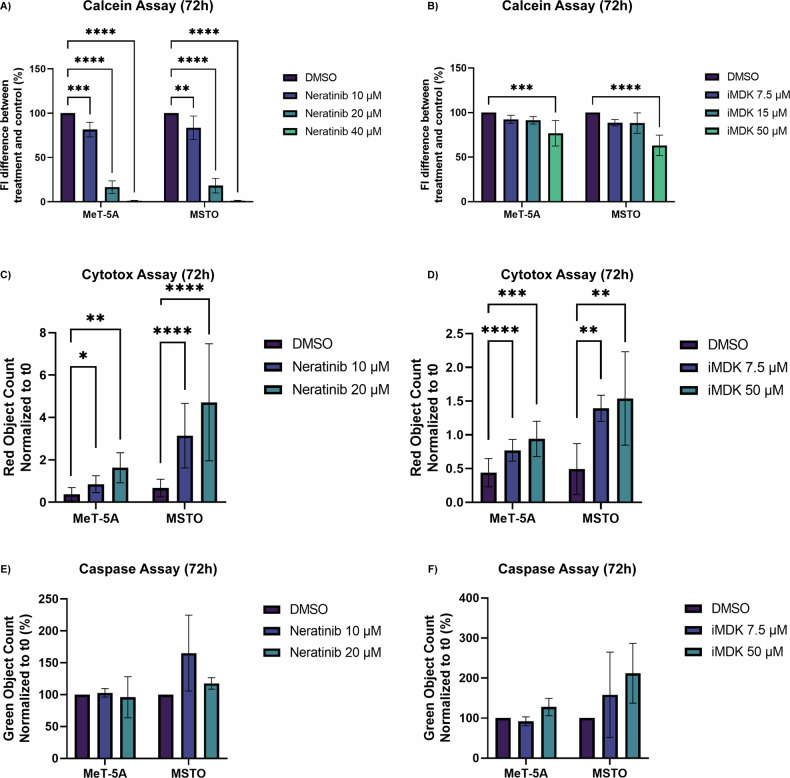
Quantitative treatment efficacy of Neratinib and iMDK. **A** Fluorescence intensity (%) of the cell lines 72 h after the treatment with Neratinib at increasing doses (10, 20, 40 µM). **B** Fluorescence intensity (%) of the cell lines 72 h after the iMDK treatment at increasing doses (7.5, 15, 50 µM). Red object count for Neratinib (**C**) and iMDK (**D**) treatment showing cytotoxicity. Green object count for Neratinib (**E**) and iMDK (**F**) treatment. These overview bar graphs indicate that overall, the expression of caspase 3/7 has not increased significantly when compared to relevant controls. The vehicle control (DMSO) is indicated in purple. MSTO and MeT-5A treated cells are indicated in blue and green. ANOVA test; *p* value 0.0332(*), 0.0021(**), 0.0002(***), <0.0001(****).

Following a literature search, no specific drugs were available for inhibiting BAG2 and MAD2L1. Only an experimental molecule (iMDK) has been developed as a specific inhibitor of MDK (approval has not been granted for this molecule, yet) [22, 23]. For MAD2L1, we identified a drug (Neratinib) approved for inhibiting HER1 and HER2 [24–26]. However, also an activity on MAD2L1 has been detected in more recent times [27]. Keeping in mind these limitations, we explored the activities of iMDK and Neratinib on MPM. First, we assessed Neratinib and iMDK for their potential inhibitory effects on the viability of MeT-5A and MSTO by using a calcein assay. FI was measured 72 h after the administration of drugs. Treatment with increasing concentrations of Neratinib (10 up to 40 µM) for 72 h produced a dose-dependent reduction in the proliferation of both cell lines compared to the control (DMSO) (Fig. 5A). On the other hand, iMDK was effective on both cell lines only at the highest dose (50 µM) (Fig. 5B). Therefore, for the next experiments we employed two doses for both drugs: 10 and 20 µM for Neratinib and 7.5 and 50 µM for iMDK. Then, cytotoxicity was measured with Cytotox Red Dye. As shown in Fig. 5C, D, a statistically significant increase in the rate of dead cells in the treated cell cultures was observed as compared to controls and this increase was dose-dependent in both cell lines.

**Fig. 6:**
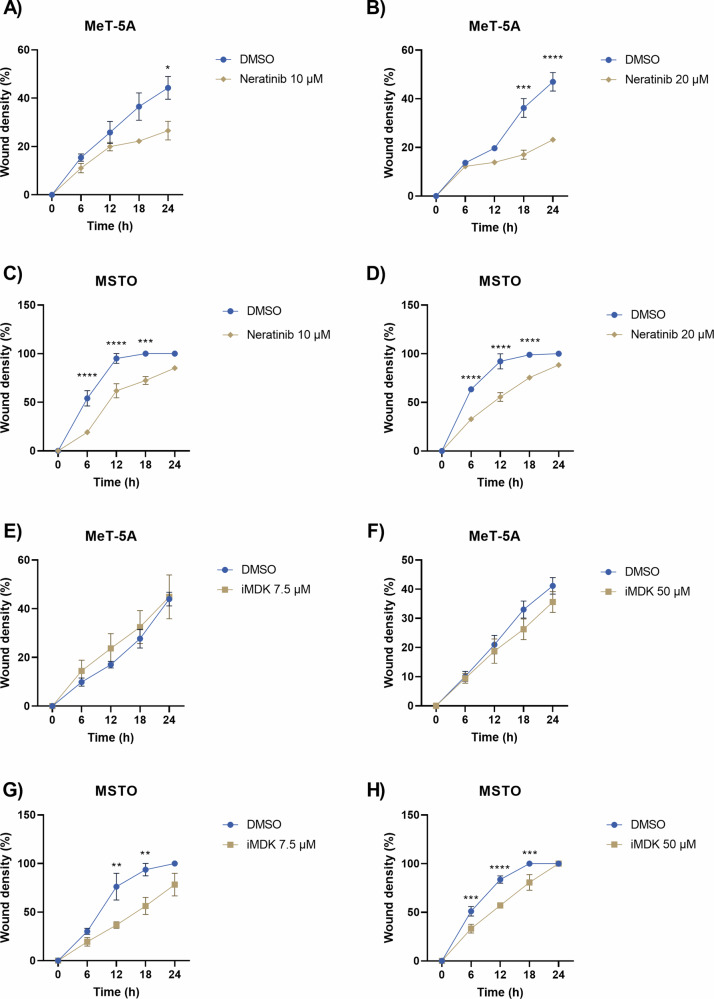
Time course analysis of the wound closure. **A**, **B** MeT-5A and **C**, **D** MSTO cells were treated just with DMSO (in blue), or with Neratinib (10 or 20 µM, in yellow). **E**, **F** MeT-5A and **G**, **H** MSTO cells were treated just with DMSO (in blue), or with iMDK (7.5 or 50 µM, in yellow). Mann–Whitney test; *p* value 0.0332(*), 0.0021(**), 0.0002(***), <0.0001(****).

For assessing apoptosis, cells were treated with Neratinib or iMDK in combination with the specific probe for caspase 3/7 activation and a statistically not significant increase of caspase 3/7 activation was observed in MSTO cell line at the highest dose (Fig. 5E, F). Finally, the wound healing assay showed that Neratinib strongly impaired the mobility of both cell lines at both doses within 24 h, while iMDK induced a reduced mobility only of MSTO cell lines statistically significant already after 12 h of treatment (Fig. 6).

As HER2 is one of the main targets of Neratinib [28], we wanted to ensure that the effects seen on MPM cells were actually due to the targeting of MAD2L1 and not a consequence of Neratinib’s effects on HER2. First, we accessed TCGA and found that HER2 is not overexpressed in MPM (Supplementary Fig. S4). Then, we verified the HER2 expression in MSTO cell line by accessing DepMap database and with an immunocytochemistry analysis. All these analyses strongly suggested that MPM in general, and the MSTO cell line in particular, do not express HER2 (Supplementary Fig. S5B). Another potential target of Neratinib is the EGFR or HER1. Therefore, as for HER2, we wanted to ensure that the effects seen on MPM cells were not to be ascribed to EGFR-targeting. For this purpose, MeT-5A and MSTO were subjected to EGFR gene silencing (Supplementary Fig. S6), either alone or in combination with Neratinib (10 or 20 µM). As shown in Supplementary Fig. S7A–C, the results under Neratinib were the same, irrespectively whether cells were co-administered with the control-siRNA or EGFR-siRNA. This corroborated the idea that the observed effects should not be ascribed to the targeting of EGFR.

### In summary, are MDK, BAG2, and MAD2L1 bona fide CDGs?

**Fig. 7:**
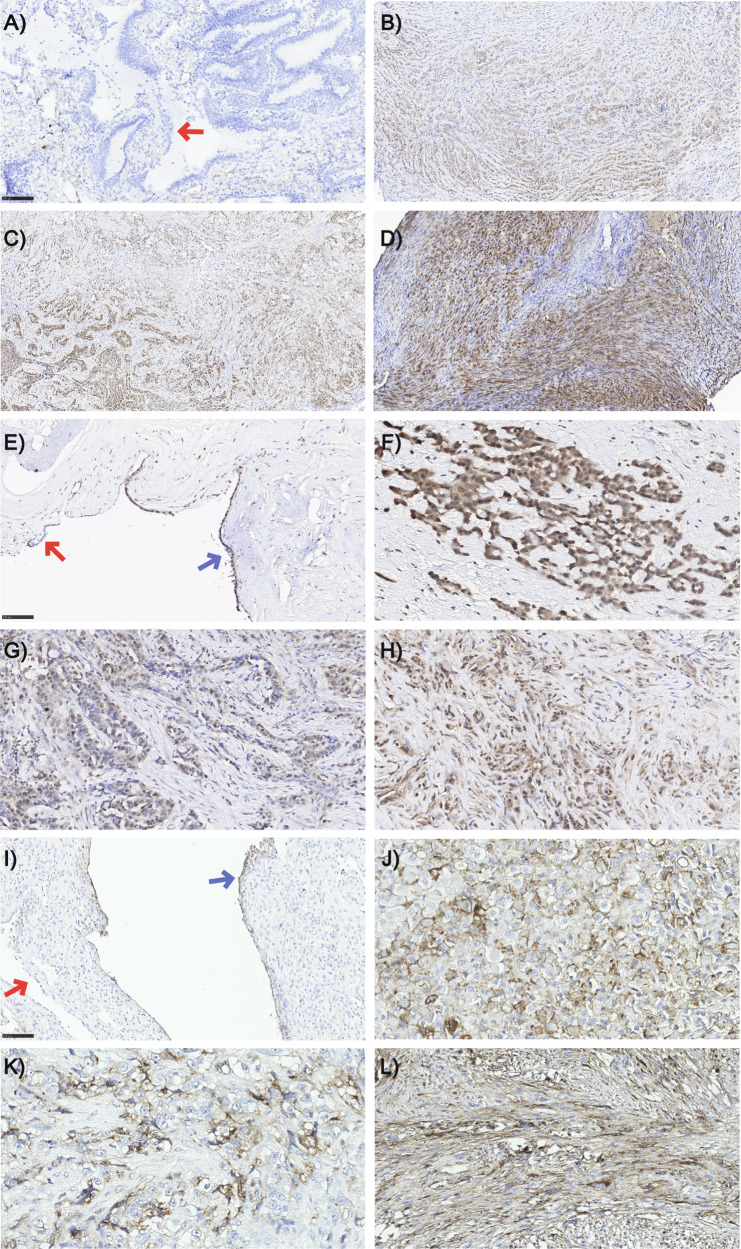
Representative immunohistochemical staining of MPM samples showing intense expression of MDK, MAD2L1, and BAG2 proteins in tumor cells (brown staining). **A**–**D** Representative BAG2 immunostainings in RMP, EMM, BMM, and SMM, respectively. Moderate to strong expression in the three types of MPM, while no expression is visible in the hyperplastic mesothelium in RMP (red arrow). **E**–**H** Representative MAD2L1 immunostainings in RMP, EMM, BMM, and SMM, respectively. Strong expression is present in the three types of MPM, but there are areas of discernable expression in the mesothelial cells on the pleural surface in RMP as well (blue arrow positive mesothelial area, red arrow negative area). **I**–**L** Representative MDK immunostainings in RMP, EMM, BMM, and SMM, respectively. Strong expression is seen in the three types of MPM; however, some focal expression is also detectable in the mesothelial cells on the pleural surface in RMP (blue arrow focal staining, red arrow negative staining). Magnification: RMPs ×400, MPMs ×200.

**Fig. 8:**
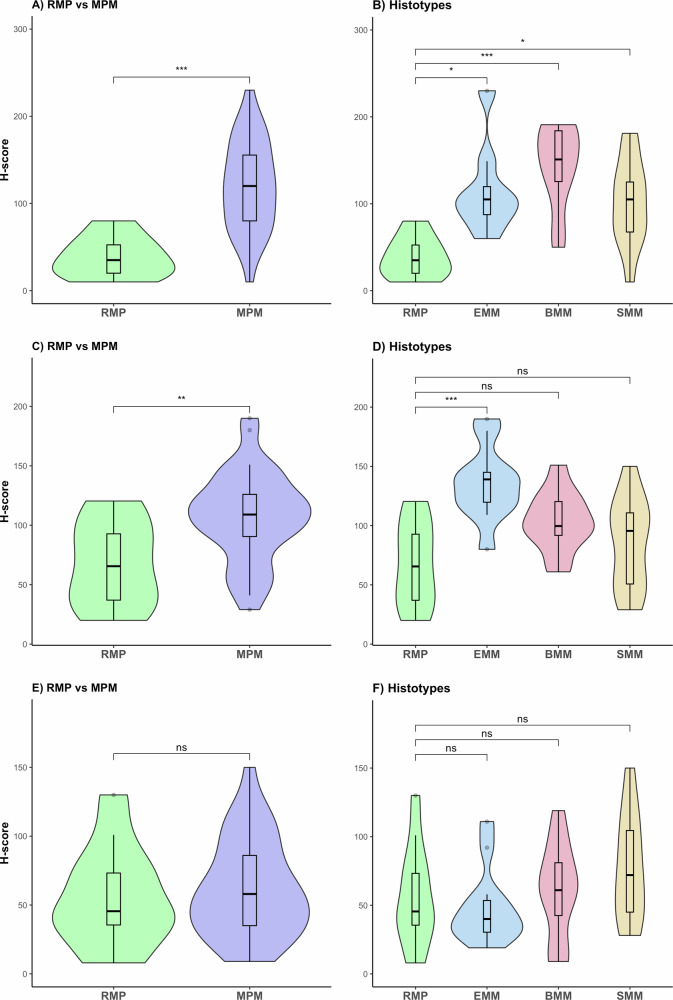
Quantification of protein expression in RMP vs MPM as assessed by IHC and H-score. Graphs for BAG2 immunostaining in RMP vs MPM regardless of subtype (**A**) and RMP vs the different subtypes of MPM (**B**), respectively. Graphs for MAD2L1 immunostaining in RMP vs MPM regardless of subtype (**C**) and RMP vs the different subtypes of MPM (**D**), respectively. Graphs for MDK immunostaining in RMP vs MPM regardless of subtype (**E**) and RMP vs the different subtypes of MPM (**F**), respectively. Kruskal–Wallis test followed by a post hoc Dunn’s test with Bonferroni correction was used to evaluate the statistical difference between the median of each group, *p* value <0.001(***), <0.01(**), <0.05(*).

BAG2, MAD2L1, and MDK were further studied on FFPE tissues derived from patients with MPM or RMP. Sections were immunoassayed with specific Abs against these proteins and scored (Fig. 7). In addition, an adjacent tissue section from each specimen was stained with hematoxylin and eosin (Histolab Products AB, Gothenburg, Sweden) for histological reference (data not shown). As summarized in the graphs (Fig. 8), considering biopsies and resections together, there was a statistically significant difference of BAG2 and MAD2L1 expression levels in tumor tissues as compared to RMP (Fig. 8A, C). In detail, despite an inter-patient variability, BAG2 showed a statistically significant higher expression in all the subtypes of MPM (Fig. 8B) when compared to RMP, while a statistically significant overexpression of MAD2L1 was identified only in EMMs (Fig. 8D). For a better description of these phenomena, we analyzed the AUC and the sensitivity/specificity of these proteins in their ability to detect MPM vs RMP. When analyzing RMP vs all types of MPM, BAG2 showed an AUC of 0.883 and a Yuden index of 0.77 (sensitivity = 0.77, specificity = 1) with a threshold score of 60. MAD2L1 showed an AUC of 0.797 and a Yuden index of 0.45 (sensitivity = 0.69; specificity = 0.77) with a threshold score of 90. MDK was expressed in all tissues, including RMP, and we did not observe any statistically significant difference between groups (Fig. 8E, F). The findings robustly validated prior transcriptome-level analyses and further solidified the idea that heightened protein expression aligns with the advancement of the tumor.

## Discussion

Here, first, we confirmed 14 of 15 DEGs already described [8] on an independent dataset of MPM patients. Then genes were confirmed to be inversely correlated with the prognosis. Of note, among them, we identified *UHRF1* and *SPARC*, that represent a “positive-control” of our approach, as they have been thoroughly studied and proved to be CDGs for MPM. Following preliminary silencing and functional assays on MeT-5A and MSTO cells, in the present study, we focused on *BAG2*, *MAD2L1*, and *MDK*, leaving the other genes to future studies.

In vitro gene depletion of *BAG2* and *MAD2L1* caused a significantly reduced cell growth and the activation of caspases 3/7. Despite the in vitro experiments being performed in a unique cell line (favorable because it recapitulated all the overexpressed genes) these results supported the other findings. Thus, the overexpression of these genes in MPM tissues at transcription level, the inverse correlation between transcriptome and OS, the effects of gene knock-down in the cellular model, and, last but not least, the frank higher protein overexpression at IHC level in MPM compared to RMP, led to conclude that *BAG2* and *MAD2L1* are strong candidates as drivers of pleural tumorigenesis.

Of note, the high protein expression of BAG2 and MAD2L1 in MPM vs RMP should have compensated possible selection biases introduced during the first steps of DEGs identification, when normal pleural tissues could not be employed as a reference, given their unavailability in the investigated databases.

BAG2 is a member of the Bcl-2-associated athanogene (BAG) proteins family whose biological activities are elicited through interaction with pivotal targets such as the heat-shock protein 70 [29] or p53 [30]. In particular, the overexpression of BAG2 in tumors causes the accumulation of mutant p53 leading to reduced apoptosis, and increased proliferation and metastasis [30]. To our knowledge, the present study is the first to report the role of BAG2 in MPM. Unfortunately, we could not find any specific BAG2 inhibitor commercially available.

MAD2L1 (MAD2 mitotic arrest deficient-like 1) is a member of the mitotic-spindle assembly checkpoint pathway [31]. Its disruption is associated, through mechanisms not yet fully understood, with chromosomal instability and aneuploidy [32] in MPM [33]. Neratinib ((2E)-N-[4-[[3-Chloro-4-(2-pyridinylmethoxy)phenyl]amino]-3-cyano-7-ethoxy-6-quinolinyl]-4-(dimethylamino)-2-butenamide) could be employed as inhibitor of MAD2L1. Initially, this TKI small molecule showed a high affinity for proteins of the HER family. Thus, it was clinically approved in 2017 as adjuvant therapy targeting HER2 in HER2-positive early-stage breast cancers who have completed adjuvant trastuzumab-based therapy [24, 34]. In 2020, the FDA also approved Neratinib for use in combination with capecitabine as a treatment option for advanced or metastatic HER2-positive breast cancer patients who have received two or more prior anti-HER2-based regimens in the metastatic setting [35]. Currently, Neratinib is undergoing clinical trials for triple-negative breast cancer (https://clinicaltrials.gov/ct2/show/NCT03812393) and it is under study for HER1-expressing (EGFR) lung adenocarcinoma [25, 26, 36], ovarian carcinoma [28, 37, 38], and biliary tract cancer [39]. However, recent studies showed that it can bind MAD2L1 with high affinity, thereby inducing conformational changes and causing its inhibition [27].

One could hypothesize that the activity of Neratinib on MSTO cells could be ascribed to the inhibition of EGFR or HER2. Indeed, EGFR is expressed in MSTO cells, as shown by our WB analysis (Supplementary Fig. S6) and reported in previous studies [40]. On the contrary, our ICC showed that HER2 is not expressed in MSTO cells, confirming what was reported in TCGA portal. Therefore, we studied whether Neratinib could be active through an EGFR-dependent mechanism. Thus, we measured the extent of cell growth, cytotoxicity, and caspase 3/7 activation when Neratinib and EGFR-siRNA pool were administered alone or in combination. We found that the activity of Neratinib was similar, irrespective of the silencing status of EGFR, ruling out the hypothesis of an EGFR-dependent mechanism. These data further strengthen the idea that in these cells Neratinib may act by affecting MAD2L1 activity.

However, EGFR is overexpressed in a high percentage (50–70%) of MPM patients, and a cancer-driving role of EGFR has been already shown for this cancer [41–43]. Therefore, it is reasonable to hypothesize that Neratinib could be of benefit by inhibiting both MAD2L1 and EGFR, fully justifying its repurposing for MPM patients.

Concerning *MDK*, in silico analyses and in vitro experiments suggested MDK as a likely CDG. MDK is a heparin-binding growth factor expressed in a variety of tissues, including the nervous system, kidney, liver, lung, and gastrointestinal tract [44]. MDK is highly expressed during embryogenesis, and it has been involved in inflammation and neurological disorders [45, 46]. However, of note, MDK interacts with a variety of plasma membrane receptors controlling also many cancer-related behaviors [47]. Elevated levels of MDK have been detected in blood and tissues of patients with several types of tumors, making MDK a potential cancer biomarker [48]. In MPM, the *MDK* promoter was found to be highly activated [49]. The inhibition of MDK is under consideration as a strategy for cancer therapy, and an inhibitor (iMDK) has been developed as an experimental molecule currently used in preclinical models [22, 23]. This molecule showed a marked activity against malignant cell lines from several types of tumors, both in vitro and in vivo [50]. Following the administration of iMDK, mice showed a strong response against xenografted oral squamous cell carcinoma cell lines resulting in a dose-dependent inhibition of proliferation [22]. Moreover, iMDK treatment induced a decrease in migration of endothelial cells and an antitumoral response against NSCLC xenografted cells in an in vivo orthotopic mouse model. iMDK showed also similar responses in a rodent model of spontaneous lung metastasis where it inhibited the metastatic process [23].

In the present study, we found that in vitro treatments with iMDK caused a significant reduction in cell growth, in part phenocopying what was observed with specific gene silencing. However, there was no observed activation of the caspase 3/7 pathway, suggesting that the reduction in proliferation may not be due to apoptosis. Rather, treated cells exhibited an increase in cytotoxicity suggesting that iMDK could induce non-apoptotic cell death. The fact that MDK was highly expressed at IHC level also in RMP led us to hypothesize that this protein could be involved in tumor progression as well as in a more general mechanism of proliferation of mesotheliocytes, likely induced by inflammatory conditions.

The present study has the limitation that in vitro experiments were performed only in MSTO cells. However, we screened several cell lines, as reported in ref. [8], and the simultaneous overexpression of the three targets was found only in these cells. Thus, we used only MSTO cells for a quick and simple proof-of-concept on the putative effects occurring following the inhibition of BAG2, MAD2L1, or MDK in a model of malignant cells where all of them were overexpressed.

In any case, *BAG2* and *MAD2L1* showed all the features for being considered CDGs and, when evaluated as potential biomarkers for MPM, they also showed to be promising for differentially defining MPM from RMP. Moreover, these findings also led us to detect an already approved drug (Neratinib) that conceivably has a rational for being repurposed also for MPM patients. Therefore, further investigation for the translation applicability of the present results into clinical practice is fully warranted.

## Supplementary information

Supplementary figures

## Data Availability

Publicly available datasets were analyzed in this study. These data can be found here: TCGA, project: TCGA-MESO, https://portal.gdc.cancer.gov/projects/TCGA; GEO, accession number: GSE94555, https://www.ncbi.nlm.nih.gov/geo/query/acc.cgi; EGA: https://ega-archive.org/datasets/EGAD00001001915. References are given in the text for the datasets taken from the literature on MPM. The new data presented in this study are available in the “Results” and Supplementary Materials sections. Further information is available from the corresponding author upon request.

## Code availability

The R scripts used for the analysis of differential expression and survival studies are accessible upon request.

## References

[1] Kuroda A. Recent progress and perspectives on the mechanisms underlying asbestos toxicity. Genes Environ Off J Japanese Environ Mutagen Soc. 2021;43:46. 10.1186/s41021-021-00215-0 PMC8507173 34641979

[2] Na B-S, Kim JS, Hyun K, Park IK, Kang CH, Kim YT. Outcomes of the multimodal treatment of malignant pleural mesiothelioma: the role of surgery. Korean J Thorac Cardiovasc Surg. 2018;51:35–40. 29430427 10.5090/kjtcs.2018.51.1.35 PMC5796616

[3] Vogelzang NJ, Rusthoven JJ, Symanowski J, Denham C, Kaukel E, Ruffie P, et al. Phase III study of pemetrexed in combination with cisplatin versus cisplatin alone in patients with malignant pleural mesothelioma. J Clin Oncol. 2003;21:2636–44. 12860938 10.1200/JCO.2003.11.136

[4] Zalcman G, Mazieres J, Margery J, Greillier L, Audigier-Valette C, Moro-Sibilot D, et al. Bevacizumab for newly diagnosed pleural mesothelioma in the Mesothelioma Avastin Cisplatin Pemetrexed Study (MAPS): a randomised, controlled, open-label, phase 3 trial. Lancet. 2016;387:1405–14. 26719230 10.1016/S0140-6736(15)01238-6

[5] Wadowski B, Bueno R, De Rienzo A. Immune microenvironment and genetics in malignant pleural mesothelioma. Front Oncol. 2021;11. 10.3389/fonc.2021.684025. 10.3389/fonc.2021.684025 PMC8226027 34178677

[6] Gray SG, Mutti L. Immunotherapy for mesothelioma: a critical review of current clinical trials and future perspectives. Transl Lung Cancer Res. 2020;9:S100–S119. 32206576 10.21037/tlcr.2019.11.23 PMC7082257

[7] Perrino M, De Vincenzo F, Cordua N, Borea F, Aliprandi M, Santoro A, et al. Immunotherapy with immune checkpoint inhibitors and predictive biomarkers in malignant mesothelioma: work still in progress. Front Immunol. 2023;14:1121557. 36776840 10.3389/fimmu.2023.1121557 PMC9911663

[8] Morani F, Bisceglia L, Rosini G, Mutti L, Melaiu O, Landi S, et al. Identification of overexpressed genes in malignant pleural mesothelioma. Int J Mol Sci. 2021;22. 10.3390/ijms22052738. 10.3390/ijms22052738 PMC7962966 33800494

[9] Andrews S. FastQC: a quality control tool for high throughput sequence data. 2010. http://www.bioinformatics.babraham.ac.uk/projects/fastqc/.

[10] Dobin A, Davis CA, Schlesinger F, Drenkow J, Zaleski C, Jha S, et al. STAR: ultrafast universal RNA-seq aligner. Bioinformatics. 2013;29:15–21. 23104886 10.1093/bioinformatics/bts635 PMC3530905

[11] Anders S, Pyl PT, Huber W. HTSeq-a Python framework to work with high-throughput sequencing data. Bioinformatics. 2015;31:166–9. 25260700 10.1093/bioinformatics/btu638 PMC4287950

[12] Durinck S, Spellman PT, Birney E, Huber W. Mapping identifiers for the integration of genomic datasets with the R/Bioconductor package biomaRt. Nat Protoc. 2009;4:1184–91. 19617889 10.1038/nprot.2009.97 PMC3159387

[13] Love MI, Huber W, Anders S. Moderated estimation of fold change and dispersion for RNA-seq data with DESeq2. Genome Biol. 2014;15:550. 25516281 10.1186/s13059-014-0550-8 PMC4302049

[14] Wickham H. ggplot2: elegant graphics for data analysis. New York: Springer-Verlag; 2016. https://ggplot2.tidyverse.org.

[15] Çakılkaya P, Sørensen RR, Jürgensen HJ, Krigslund O, Gårdsvoll H, Nielsen CF, et al. The collagen receptor uPARAP in malignant mesothelioma: a potential diagnostic marker and therapeutic target. Int J Mol Sci. 2021;22:11452. 34768883 10.3390/ijms222111452 PMC8583732

[16] Detre S, Saclani Jotti G, Dowsett M. A ‘quickscore’ method for immunohistochemical semiquantitation: validation for oestrogen receptor in breast carcinomas. J Clin Pathol. 1995;48:876–8. 7490328 10.1136/jcp.48.9.876 PMC502883

[17] Zimling ZG, Sørensen JB, Gerds TA, Bech C, Andersen CB, Santoni-Rugiu E. Low ERCC1 expression in malignant pleural mesotheliomas treated with cisplatin and vinorelbine predicts prolonged progression-free survival. J Thorac Oncol. 2012;7:249–56. 22031231 10.1097/JTO.0b013e318233d6a9

[18] Sage A, Martinez V, Minatel B, Pewarchuk M, Marshall E, MacAulay G, et al. Genomics and epigenetics of malignant mesothelioma. High-Throughput. 2018;7:20. 30060501 10.3390/ht7030020 PMC6163664

[19] Sinn K, Mosleh B, Hoda MA. Malignant pleural mesothelioma: recent developments. Curr Opin Oncol. 2021;33:80–6. 33186182 10.1097/CCO.0000000000000697

[20] Reardon ES, Shukla V, Xi S, Gara SK, Liu Y, Straughan D, et al. UHRF1 is a novel druggable epigenetic target in malignant pleural mesothelioma. J Thorac Oncol. 2021;16:89–103. 32927122 10.1016/j.jtho.2020.08.024 PMC7775915

[21] Ollila H, Paajanen J, Wolff H, Ilonen I, Sutinen E, Välimäki K, et al. High tumor cell platelet-derived growth factor receptor beta expression is associated with shorter survival in malignant pleural epithelioid mesothelioma. J Pathol Clin Res. 2021;7:482–94. 33955203 10.1002/cjp2.218 PMC8363931

[22] Masui M, Okui T, Shimo T, Takabatake K, Fukazawa T, Matsumoto K, et al. Novel midkine inhibitor iMDK inhibits tumor growth and angiogenesis in oral squamous cell carcinoma. Anticancer Res. 2016;36:2775–81. 27272788

[23] Shin DH, Jo JY, Kim SH, Choi M, Han C, Choi BK, et al. Midkine is a potential therapeutic target of tumorigenesis, angiogenesis, and metastasis in non-small cell lung cancer. Cancers. 2020;12:2402. 32847073 10.3390/cancers12092402 PMC7563676

[24] Lüftner D, Tesch H, Schmidt M, Hartkopf AD, Streicher S, Resch A, et al. Neratinib as extended adjuvant therapy in patients with copositive early breast cancer: German health technology assessment-driven analyses from the ExteNET study. Eur J Cancer. 2021;150:268–77. 33971386 10.1016/j.ejca.2021.03.045

[25] Castellano GM, Aisner J, Burley SK, Vallat B, Yu HA, Pine SR, et al. A novel acquired exon 20 EGFR M766Q mutation in lung adenocarcinoma mediates osimertinib resistance but is sensitive to neratinib and poziotinib. J Thorac Oncol. 2019;14:1982–8. 31254668 10.1016/j.jtho.2019.06.015 PMC8080261

[26] Tiwari SR, Mishra P, Abraham J. Neratinib, a novel HER2-targeted tyrosine kinase inhibitor. Clin Breast Cancer. 2016;16:344–8. 27405796 10.1016/j.clbc.2016.05.016

[27] Zhou G, Zhao M, Liang R, Xie J, Chen X, Chen Q, et al. A study of the mechanism of binding between neratinib and MAD2L1 based on molecular simulation and multi-spectroscopy methods. Curr Pharm Des. 2020;25:4287–95. 10.2174/1381612825666191107102413 31696805

[28] Menderes G, Bonazzoli E, Bellone S, Black JD, Lopez S, Pettinella F, et al. Efficacy of neratinib in the treatment of HER2/neu-amplified epithelial ovarian carcinoma in vitro and in vivo. Med Oncol. 2017;34:91. 28397106 10.1007/s12032-017-0956-8 PMC5896014

[29] Qin L, Guo J, Zheng Q, Zhang H. BAG2 structure, function and involvement in disease. Cell Mol Biol Lett. 2016;21:18. 28536620 10.1186/s11658-016-0020-2 PMC5415834

[30] Yue X, Zhao Y, Liu J, Zhang C, Yu H, Wang J, et al. BAG2 promotes tumorigenesis through enhancing mutant p53 protein levels and function. Elife. 2015;4. 10.7554/eLife.08401. 10.7554/eLife.08401 PMC4561369 26271008

[31] Pulverer B. Spindle checkpoint protein links Rb pathway to aneuploidy. Nat Cell Biol. 2004;6:806. 15340446 10.1038/ncb0904-806

[32] Li Y, Benezra R. Identification of a human mitotic checkpoint gene: hsMAD2. Science. 1996;274:246–8. 8824189 10.1126/science.274.5285.246

[33] Busacca S, O’Regan L, Singh A, Sharkey AJ, Dawson AG, Dzialo J, et al. BRCA1/MAD2L1 deficiency disrupts the spindle assembly checkpoint to confer vinorelbine resistance in mesothelioma. Mol Cancer Ther. 2021;20:379–88. 33158996 10.1158/1535-7163.MCT-20-0363

[34] Wulfkuhle JD, Yau C, Wolf DM, Vis DJ, Gallagher RI, Brown-Swigart L, et al. Evaluation of the HER/PI3K/AKT family signaling network as a predictive biomarker of pathologic complete response for patients with breast cancer treated with neratinib in the I-SPY 2 TRIAL. JCO Precis Oncol. 2018;2. 10.1200/PO.18.00024. 10.1200/PO.18.00024 PMC7446527 32914002

[35] Saura C, Oliveira M, Feng Y-H, Dai M-S, Chen S-W, Hurvitz SA, et al. Neratinib plus capecitabine versus lapatinib plus capecitabine in HER2-positive metastatic breast cancer previously treated with ≥2 HER2-directed regimens: phase III NALA trial. J Clin Oncol. 2020;38:3138–49. 32678716 10.1200/JCO.20.00147 PMC7499616

[36] Sequist LV, Besse B, Lynch TJ, Miller VA, Wong KK, Gitlitz B, et al. Neratinib, an irreversible Pan-ErbB receptor tyrosine kinase inhibitor: results of a phase II trial in patients with advanced non–small-cell lung cancer. J Clin Oncol. 2010;28:3076–83. 20479403 10.1200/JCO.2009.27.9414

[37] Han C, McNamara B, Bellone S, Harold J, Manara P, Hartwich TMP, et al. The Poly (ADP-ribose) polymerase inhibitor olaparib and pan-ErbB inhibitor neratinib are highly synergistic in HER2 overexpressing epithelial ovarian carcinoma in vitro and in vivo. Gynecol Oncol. 2023;170:172–8. 36706643 10.1016/j.ygyno.2023.01.015 PMC10023457

[38] Wang S, Zhang J, Wang T, Ren F, Liu X, Lu Y, et al. Endocytic degradation of ErbB2 mediates the effectiveness of neratinib in the suppression of ErbB2-positive ovarian cancer. Int J Biochem Cell Biol. 2019;117:105640. 31689531 10.1016/j.biocel.2019.105640

[39] Harding JJ, Piha-Paul SA, Shah RH, Murphy JJ, Cleary JM, Shapiro GI, et al. Antitumour activity of neratinib in patients with HER2-mutant advanced biliary tract cancers. Nat Commun. 2023;14:630. 36746967 10.1038/s41467-023-36399-y PMC9902444

[40] Yang L, Fang H, Jiang J, Sha Y, Zhong Z, Meng F. EGFR-targeted pemetrexed therapy of malignant pleural mesothelioma. Drug Deliv Transl Res. 2022;12:2527–36. 34802094 10.1007/s13346-021-01094-2

[41] Destro A, Ceresoli GL, Falleni M, Zucali PA, Morenghi E, Bianchi P, et al. EGFR overexpression in malignant pleural mesothelioma: an immunohistochemical and molecular study with clinico-pathological correlations. Lung Cancer. 2006;51:207–15. 16384623 10.1016/j.lungcan.2005.10.016

[42] Okuda K, Sasaki H, Kawano O, Yukiue H, Yokoyama T, Yano M, et al. Epidermal growth factor receptor gene mutation, amplification and protein expression in malignant pleural mesothelioma. J Cancer Res Clin Oncol. 2008;134:1105–11. 18392851 10.1007/s00432-008-0384-4 PMC12161759

[43] Agarwal V, Lind MJ, Cawkwell L. Targeted epidermal growth factor receptor therapy in malignant pleural mesothelioma: where do we stand? Cancer Treat Rev. 2011;37:533–42. 21183281 10.1016/j.ctrv.2010.11.004

[44] Muramatsu T. Structure and function of midkine as the basis of its pharmacological effects. Br J Pharmacol. 2014;171:814–26. 23992440 10.1111/bph.12353 PMC3925020

[45] Choudhuri R, Zhang HT, Donnini S, Ziche M, Bicknell R. An angiogenic role for the neurokines midkine and pleiotrophin in tumorigenesis. Cancer Res. 1997;57:1814–9. 9135027

[46] Zhang Z-Z, Wang G, Yin S-H, Yu X-H. Midkine: a multifaceted driver of atherosclerosis. Clin Chim Acta. 2021;521:251–7. 34331952 10.1016/j.cca.2021.07.024

[47] Filippou PS, Karagiannis GS, Constantinidou A. Midkine (MDK) growth factor: a key player in cancer progression and a promising therapeutic target. Oncogene. 2020;39:2040–54. 31801970 10.1038/s41388-019-1124-8

[48] Jones DR. Measuring midkine: the utility of midkine as a biomarker in cancer and other diseases. Br J Pharmacol. 2014;171:2925–39. 24460734 10.1111/bph.12601 PMC4055197

[49] Kubo S, Kawasaki Y, Yamaoka N, Tagawa M, Kasahara N, Terada N, et al. Complete regression of human malignant mesothelioma xenografts following local injection of midkine promoter-driven oncolytic adenovirus. J Gene Med. 2010;12:681–92. 20635326 10.1002/jgm.1486 PMC2938764

[50] Hao H, Maeda Y, Fukazawa T, Yamatsuji T, Takaoka M, Bao X-H, et al. Inhibition of the growth factor MDK/midkine by a novel small molecule compound to treat non-small cell lung cancer. PLoS ONE. 2013;8:e71093. 23976985 10.1371/journal.pone.0071093 PMC3745462

