# Supplementary material for: BAG2, MAD2L1, and MDK are cancer-driver genes and candidate targets for novel therapies in malignant pleural mesothelioma
Source: Cancer Gene Ther. 2024 Sep 12;31(11):1708–20. doi: 10.1038/s41417-024-00805-4 (PMC11567880; doi:10.1038/s41417-024-00805-4)
Supplement: Supplementary file 1 — Supplementary figures [file 41417_2024_805_MOESM1_ESM.pdf]

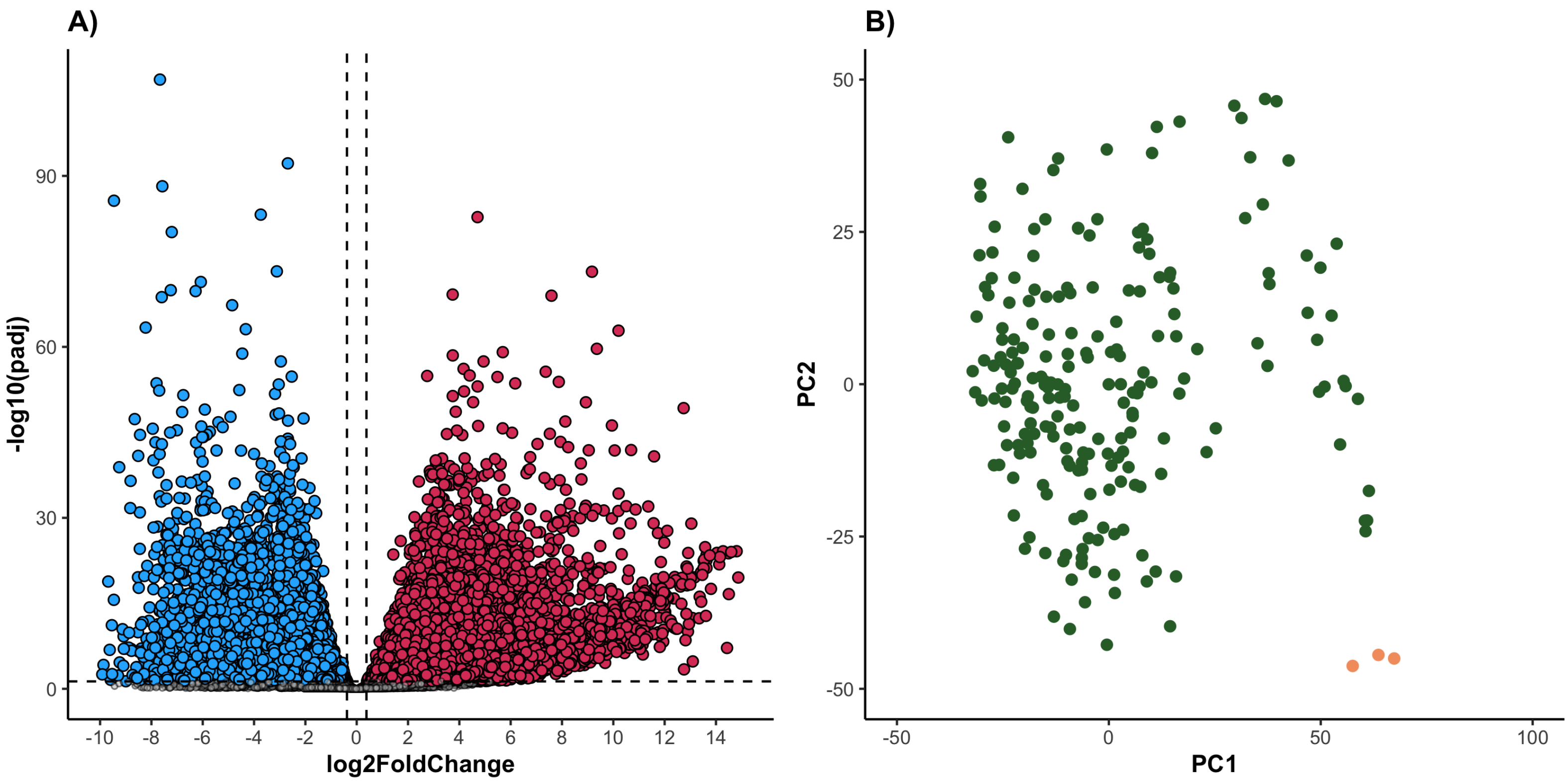

Supplementary figure S1. (A) Volcano plot for differential gene expression analysis in MPM. Genes that are downregulated are shown in blue, genes that are overexpressed are in red, while genes that are not significantly changed are shown in grey falling outside the cut off values of FDR < 0.05 and |log2fc| > 0.38. (B) Principal component analysis (PCA) plot of 3 normal lung samples (orange) and 211 MPM patients (green). Samples from MPM patients and controls were clustered individually, according to the PCA score plot.

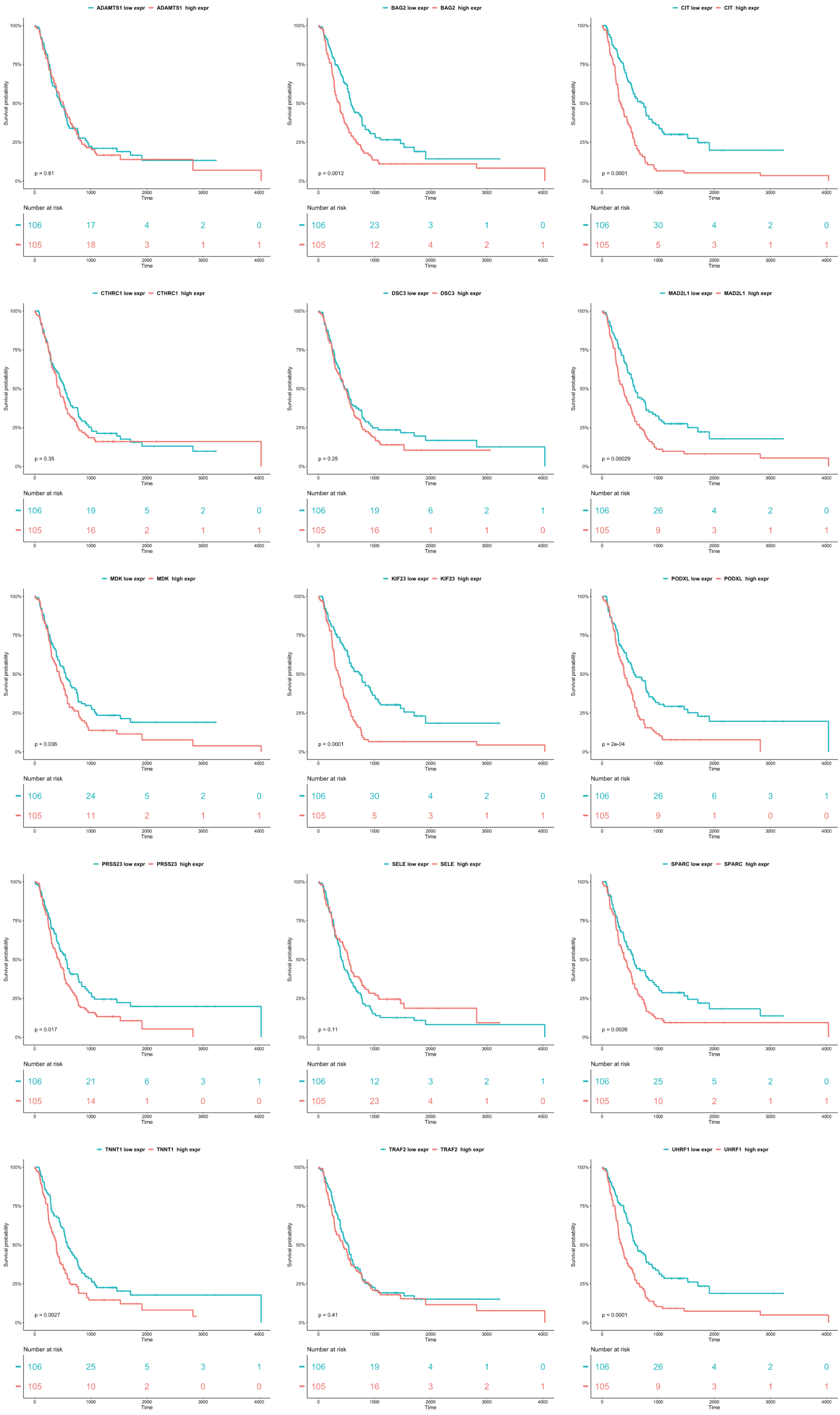

Supplementary figure S2. KM curves. Under each KM-plot there is the plot of the number of patients at risk. In red the high-expression group, in blue the low-expression group. The time is expressed in days. Log rank test was used to calculate the significant difference between the two curves.

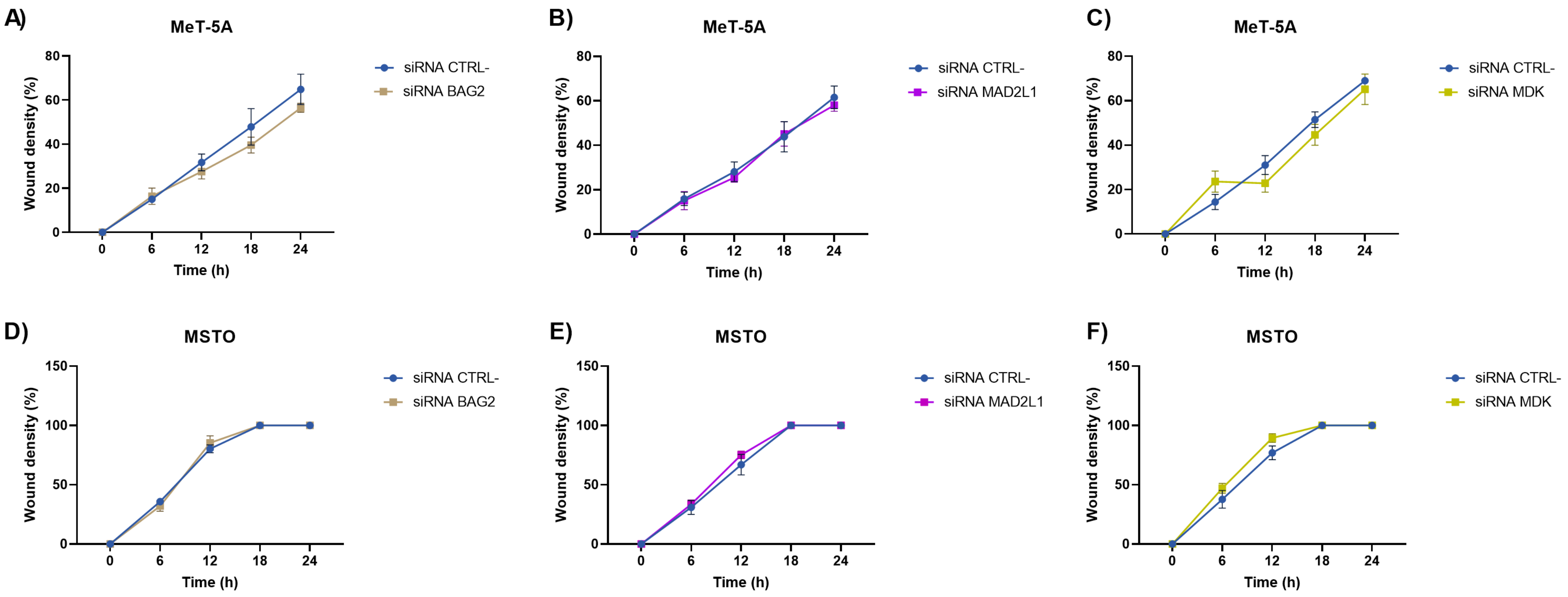

Supplementary figure S3. Time course analysis of the wound closure. (A, B, C) MeT-5A, (D, E, F) MSTO cell line. In blue the cells transfected with the negative control siRNA, while in green, brown and purple the siRNA targeting MDK, BAG2 and MAD2L1, respectively.

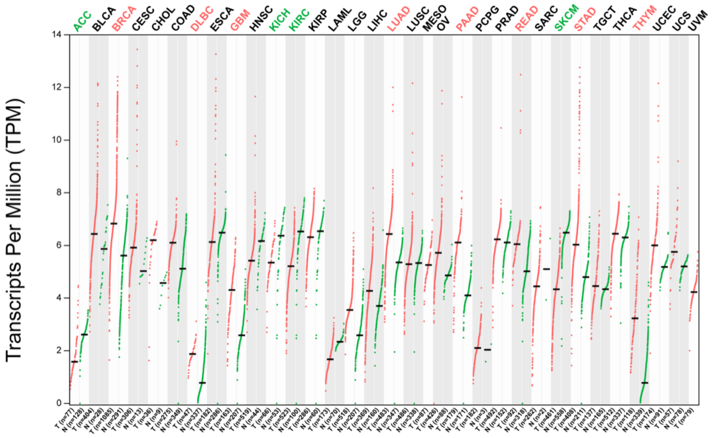

Supplementary figure S4. The gene expression profile depicts the number of HER2 transcripts per million in TCGA database. Dot plots (mRNA levels) across tumor samples are shown in red, with MESO representing MPM53. For most tumors (though not for MPM), data for their corresponding normal tissue of origin are shown in green and the median for each group is a black bar. BRCA, LUAD, DLBC, GBM, PAAD, READ, STAD, and THYM are tumors with high HER2 expression in malignant tissues as compared to non-malignant tissues. HER2 expression in malignant tissues is reduced in ACC, KICH, KIRP, and SKCM, while for the other cancer types the difference in expression with the normal counterpart is not significant.

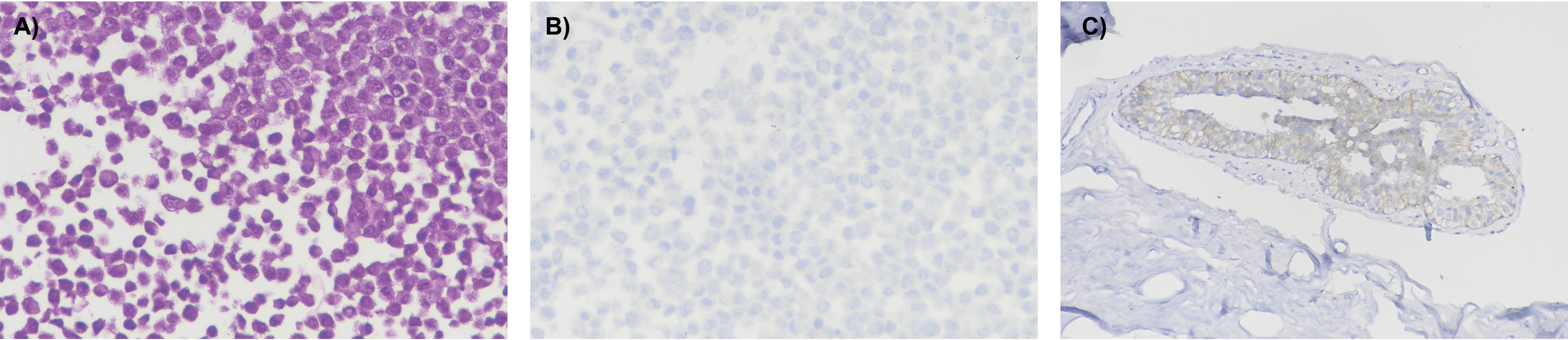

Supplementary figure S5. (A) Hematoxylin and eosin (H&E) staining of the MSTO MPM cell line, (B) immunocytochemistry staining of HER2 on MSTO cell line indicating no overexpression of this protein in the tumor cells, and (C) immunocytochemistry staining of HER2 on breast ductal carcinoma in situ tissue section, as positive control. Magnification: 40x.

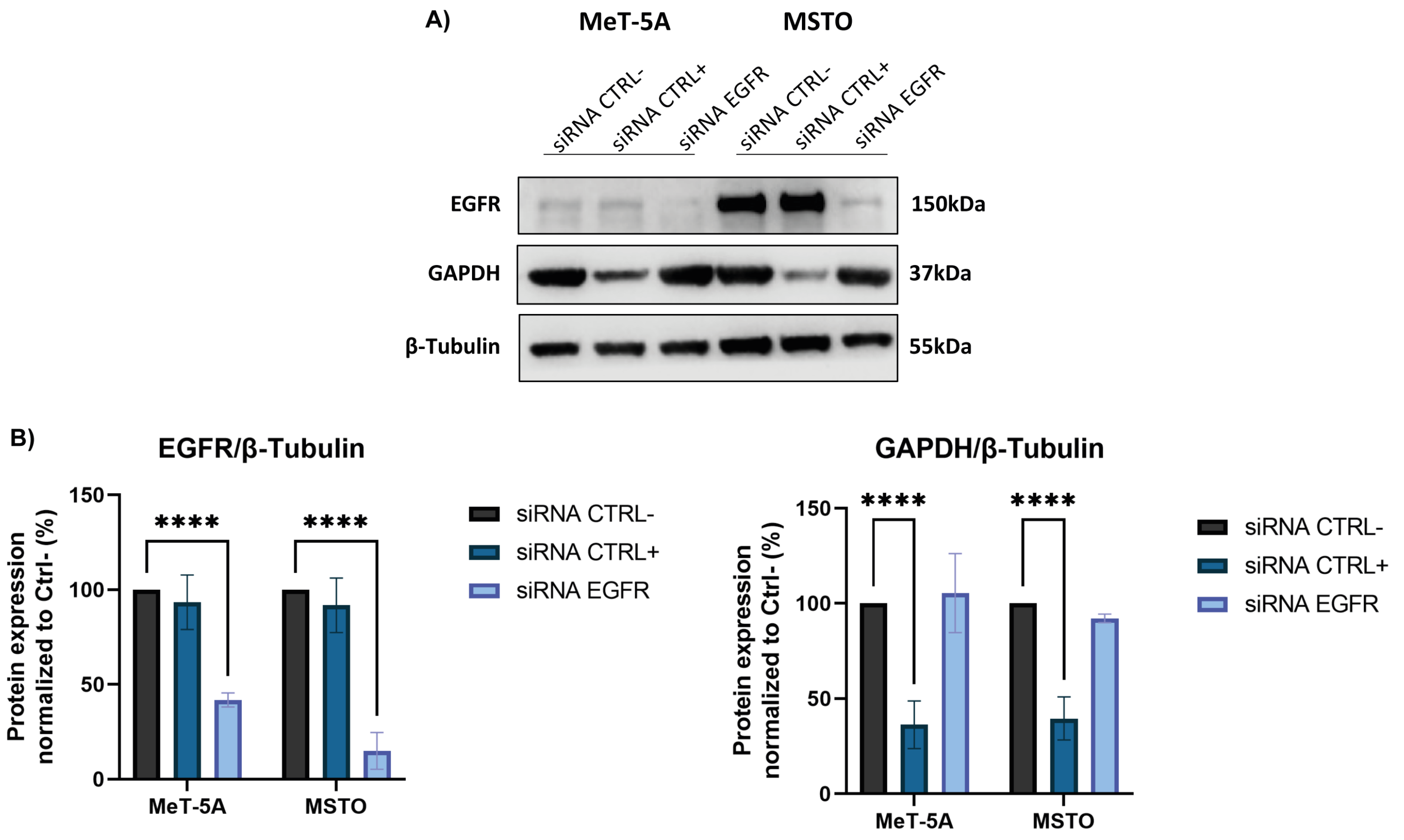

Supplementary figure S6. (A) Representative western blot of the siRNA EGFR transfection experiment. β-Tubulin was used as loading control, GAPDH was the positive control of the siRNA transfection. As shown in the blot and also in the densitometry analysis (B) there was a significant reduction of the protein levels in the silenced cells, as compared to the negative CTRL. (Mann-Whitney test; p-value < 0.05 were considered significant, p-value = 0.0332(*), 0.0021 (**), 0.0002(***), < 0.0001 (****)).

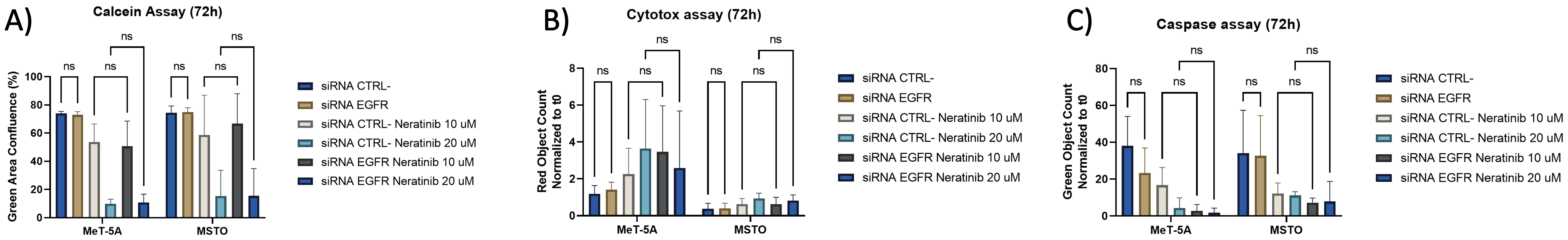

Supplementary figure S7. (A) Calcein assay, (B) Cytotox assay and (C) Caspase assay at 72 hours after silencing of EGFR or siRNA negative CTRL in MeT-5A and MSTO cells in the presence of 10 μM or 20 μM Neratinib.
